# Systematic review on the comparative effectiveness of foot orthoses in patients with rheumatoid arthritis

**DOI:** 10.1186/s13047-019-0338-x

**Published:** 2019-06-13

**Authors:** Marloes Tenten-Diepenmaat, Joost Dekker, Martijn W. Heymans, Leo D. Roorda, Thea P. M. Vliet Vlieland, Marike van der Leeden

**Affiliations:** 1 Amsterdam Rehabilitation Research Center | Reade, Amsterdam, the Netherlands; 2 Department of Rehabilitation Medicine, Amsterdam UMC, Vrije Universiteit Amsterdam, Amsterdam, the Netherlands; 3 Amsterdam Public Health research institute, Amsterdam UMC, Amsterdam, the Netherlands; 4 Department of Epidemiology and Biostatistics, Amsterdam Public Health research institute, Amsterdam University Medical Centers, Amsterdam, the Netherlands; 5 Department of Orthopaedics, Rehabilitation and Physical Therapy, Leiden University Medical Center, Leiden, the Netherlands

**Keywords:** Rheumatoid arthritis, Foot, Foot orthoses, Systematic review

## Abstract

**Background:**

Foot orthoses (FOs) are prescribed as an important conservative treatment option in patients with foot problems related to rheumatoid arthritis. However, a broad variation in FOs is used, both in clinical practice and in research. To date, there is no overview on the outcomes of the treatment with different kinds of FOs in patients with rheumatoid arthritis and a specific foot problem. The objectives of the present study were to summarize the comparative effectiveness of FOs in the treatment of various foot problems in patients with rheumatoid arthritis, on the primary outcomes foot function and foot pain, and the secondary outcomes physical functioning, health related quality of life, compliance, adverse events, the costs of FOs and patient satisfaction.

**Methods:**

Studies comparing different kinds of FOs, with a presumed therapeutic effect, in the treatment of foot problems related to rheumatoid arthritis were included. A literature search was conducted in The Cochrane Central Registry for Controlled Trials (CENTRAL), PubMed, EMBASE and PEDro up to May 18th, 2018. Data was meta-analyzed, when this was not possible qualitative data analysis was performed.

**Results:**

Ten studies were identified, with a total number of 235 patients. These studies made a comparison between different materials used (soft versus semi-rigid), types of FOs (custom-made versus ready-made; total-contact versus non-total contact), or modifications applied (metatarsal bars versus domes). Also, different techniques to construct custom-made FOs were compared (standard custom-molding techniques versus more sophisticated techniques). A medium effect for (immediate) reduction of forefoot plantar pressure was found in favor of treatment with soft FOs compared to semi-rigid FOs (SMD 0.60, 95% CI 0.07–1.14; *P* = 0.03; 28 participants). Other comparisons between FOs resulted in non-significant effects or inconclusive evidence for one kind of FOs over the other.

**Conclusions:**

Foot orthoses made of soft materials may lead to more (immediate) forefoot plantar pressure reduction compared to foot orthoses constructed of semi-rigid materials. Definitive high quality RCTs, with adequate sample sizes and long-term follow-up, are needed to investigate the comparative (cost-) effectiveness of different kinds of foot orthoses for the treatment of foot problems related to rheumatoid arthritis.

**Electronic supplementary material:**

The online version of this article (10.1186/s13047-019-0338-x) contains supplementary material, which is available to authorized users.

## Background

Foot problems are frequently identified in patients with rheumatoid arthritis (RA) [1–5]. Synovitis of foot joints, especially in the forefoot, may lead to damage and deformity of these joints [1]. Subsequently, foot pain and disability may occur resulting in a reduced quality of life [1, 6, 7]. Treatment of RA consists of systemic medication and, if necessary, additional conservative or surgical treatment.

Foot orthoses (FOs) are an important conservative treatment option for RA-related foot problems [8]. FOs can be prescribed for optimizing foot mechanics and function, or for providing cushioning and off-loading of foot structures [9–11]. In general, the aim of prescribing FOs is to reduce foot pain and to improve physical function and quality of life [9, 12–15]. FOs are placed between the plantar surface of the foot and the sole of the patient’s shoe, have a presumed therapeutic effect and are either ready- or custom-made. FOs are provided according to the individual requirements of the patient.

The effectiveness of custom-made FOs in the treatment of RA-related foot problems has been summarized in three published systematic reviews [9, 14, 16]. Two reviews reported evidence for the reduction of foot pain [9, 14], one review also found weak evidence for the reduction of forefoot plantar pressure [9]. Within these systematic reviews, the effectiveness of custom-made FOs was compared to placebo/simple FOs or no FOs.

A broad variation in FOs is used in the treatment of specific RA-related foot problems, both in clinical practice and research. FOs may have several characteristics concerning materials used (e.g. rigid or soft), type (e.g. custom-made or ready-made; contoured or non-contoured) and modifications (e.g. metatarsal domes or bars, shock-absorbing paddings) [12]. Furthermore, custom-made FOs can be constructed in different ways, e.g. by using custom molding techniques or more sophisticated CAD-CAM (computer-aided design/computer-aided manufacturing) or laser sintering systems. The characteristics of FOs prescribed may depend on the target of treatment (i.e. pressure redistribution or support, stabilization or correction of foot structures) in a specific foot region (forefoot, midfoot, rearfoot or a combination). Moreover, disease stage, the expertise of health professionals, patients’ preferences, costs, access to foot care, and national and international referral patterns can play a role in the prescription of FOs [17].

To date, there is no overview on the outcomes of the treatment with different kinds of FOs in patients with RA and a specific foot problem. In addition, there is a lack of knowledge on the costs that are related to treatment with different types of FOs. Therefore, the aim of the present review was to systematically summarize the literature on the comparative effectiveness of FOs in the treatment of various foot problems in patients with RA, on the primary outcomes foot function and foot pain, and the secondary outcomes physical functioning, health related quality of life (HRQoL), compliance, adverse events, the costs of FOs and patient satisfaction.

## Methods

### Protocol and registration

A detailed protocol for the present study has been previously published in PROSPERO (Prospero Record Registration No.: CRD42018082039). The manuscript was written in accordance with the PRISMA (Preferred Reporting Items for Systematic Reviews and Meta-Analyses) statement [18].

### Eligibility criteria

#### Types of studies

(non) Randomized controlled trials (RCT), (non) randomized controlled cross-over trials and quasi-experimental clinical trials comparing different kinds of FOs were included. Only full-text original research reports, published in English, German, French, or Dutch were included. No restrictions concerning the year of publication were used.

#### Types of participants

The study population comprised patients ≥18 years of age and diagnosed with RA, or a defined subgroup of RA patients for whom data were presented separately.

#### Type of intervention and comparisons

Studies were eligible if patients received FOs with a presumed therapeutic effect for the treatment of RA related foot problems. Studies compared different FOs characteristics (i.e. materials used, type of FOs, or modifications applied) or different construction methods for manufacturing FOs. The only difference between the interventions was related to the FOs, while shoe condition and the target of the treatment remained stable.

#### Type of outcomes

Studies were eligible if at least one of the following outcomes was assessed: foot function (i.e. plantar pressure or gait parameters), foot pain, physical functioning (performance-based or self-reported), HRQoL, compliance, adverse events, the costs of FOs, or participant satisfaction.

### Information sources, search and study selection

The following electronic databases were searched from inception to May 18th 2018: the Cochrane Central Registry for Controlled Trials (CENTRAL), PubMed, EMBASE and PEDro. Detailed search strategies are presented in Additional file 1. Each database was searched independently by two researchers (MTD and MvdL). In addition, references lists of all selected publications were checked to retrieve relevant publications which have not been found with the computerized search.

Titles or abstracts of all studies were first screened independently by two reviewers (MTD and MvdL). For each selected study, the full article was retrieved. Next, the two reviewers independently performed final selection of studies to be included in the review based on the eligibility criteria. Disagreements on inclusion were resolved by discussion between the two reviewers.

### Data collection process, data items and summary measures

Data were extracted by one reviewer (MTD) using a standardized template, and verified by a second reviewer (MvdL). From each included study, information was extracted on: authors, year of publication, study design, participant description (number of participants, setting, diagnosis, age and other clinical characteristics), description of intervention (including FOs characteristics and target of treatment for a specific foot region), longest point of follow-up, outcome measures and -if applicable- mean and standard deviations for baseline, follow-up and change scores in the outcomes, or percentages of change in the outcomes. Means were estimated from graphs, when no numerical data were supplied [19]. Disagreements or discrepancies on data extraction were resolved by discussion. If the study provided data from more than one measurement instrument, then the outcome measure most prevalent across studies was used in the analysis. For the studies in which the most prevalent outcome measure was not reported, data of the instrument highest in hierarchy was used. Based on the psychometric properties of the instruments [20] the following hierarchies (highest to lowest within the categories i-v) were applied: (i) foot function (plantar pressure): *pressure time integral, peak pressure, other instrument,* (ii) foot function (gait): *cadence, stride length, other instrument*, (iii) foot pain: *Foot Function Index subscale pain (FFI pain), Visual Analogue Scale for foot pain during walking (VAS foot pain), other instrument,* (iv) physical functioning: *Foot Function Index subscale disability (FFI disability), timed walking test, other instrument,* and (v) HRQoL: *Foot Health Status Questionnaire subscale general health (FHSQ general health), Visual Analogue Scale for general well-being (VAS general well-being), other instrument.*

### Methodological quality of individual studies

The methodological quality of included studies was assessed with the Physiotherapy Evidence Database (PEDro) scale [21]. The PEDro scale has been shown to be a valid, reliable and frequently used tool for assessing methodological quality of randomized controlled trials and clinical controlled trials [22–24]. It consists of 11 items to measure the quality of each included trial. Eight items (item 2–9) are used to assess internal validity and two items to assess interpretability of results (item 10–11). Item 1, assessing external validity, is excluded in calculating the total score [25]. Therefore, the score may range from 0 to 10 points. When a repeated measures or cross-over design was used, item 4 (similarity of baseline prognostic indicators between groups) was not applicable and the maximum possible score was 9. The score obtained for each study was divided by the maximum possible score and multiplied by 100 to provide a “study quality percentage”. Study quality percentages were then classified as high (≥55–100%), fair (≥35- < 55%), or low (< 35%) according to Teasell et al. [26].

Quality assessments were independently evaluated by two reviewers (MTD and MvdL). Disagreements were resolved by discussion and, if necessary, by consultation of the third reviewer (JD).

### Data synthesis

Data synthesis was conducted for the effect of FOs on (i) the primary outcomes foot function and foot pain and (ii) the secondary outcomes physical functioning, HRQoL, compliance, adverse events, the costs of FOs and participant satisfaction. For studies with no follow-up time, the immediate effect was used in analysis. The immediate effect reflects the differences within the same measurement session between the different FO conditions*.* Quantitative data analysis (meta-analysis) was conducted for between-group comparison of FOs characteristics or FOs construction methods. Outcomes measured during (in case of single-session measurement (studies with no follow-up)) or after wearing FOs (longitudinal studies with differing follow-up time) were used and aggregated in meta-analyses. Subgroup meta-analyses were performed in case of a sufficient number of studies for further specification, i.e. targeted foot region; follow-up time shoe condition; study quality.

Pooling of effect sizes across studies was performed using the standardized mean difference (SMD) and 95% confidence intervals (CI) in a random effects model [27]. SMDs were interpreted as 0.2 (small), 0.5 (medium) and 0.8 (large) [28]. The results are presented in forest plots for each comparison. Funnel plots were constructed for meta-analyses with ≥2 studies, to assess possible publication bias. Meta-analyses were conducted in computer software R [29]. Heterogeneity was tested using the eye ball test (forest plot).

When quantitative data analysis was not possible, a qualitative data analysis (best-evidence synthesis) was conducted. The data were summarized by assigning five levels of evidence (strong, moderate, weak, inconclusive and inconsistent) according to criteria adapted from Ariëns et al. (Table 1) [30].

**Table 1:** Strength of evidence criteria [30]

Strong	At least 2 high-quality studies with consistent findings
Moderate	1 high-quality study and at least 2 low-quality studies with consistent findings
Weak	At least 2 low-quality studies with consistent findings
Inconclusive	Insufficient or conflicting studies
Inconsistent	Agreement of findings in < 75% of studies

## Results

### Study selection

**Fig. 1:**
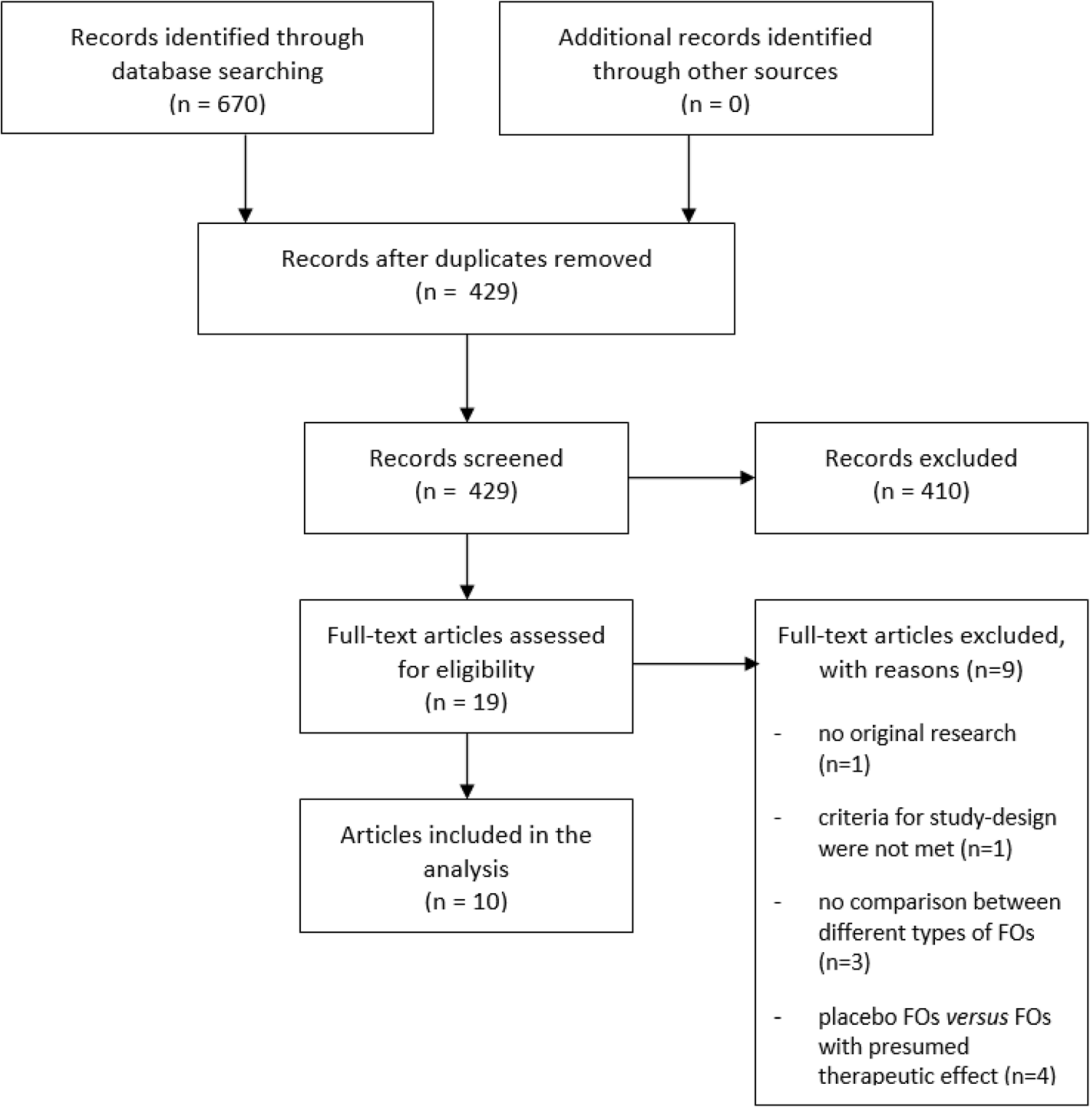
PRISMA flow diagram

The literature search resulted in a total number of 670 hits. After duplicate removal, 429 hits were screened on title or abstract. This resulted in 19 full-text articles that were studied for eligibility, of which 10 articles were included in the systematic review (Fig. 1).

### Characteristics of included studies

The included studies consisted of four RCTs [31–34] of which two with a repeated measures design [31, 32], three controlled clinical trials with a repeated measures design [11, 35, 36], one controlled cross-over trial [37], and two quasi-experimental clinical trials with a repeated measures design [38, 39]. FOs targeting forefoot problems were investigated in six studies [11, 31, 35, 36, 39]. FOs targeting hindfoot problems were investigated in one study [37]. Three studies investigated the effect of FOs without a specified region of interest [33, 34, 38]. Four studies specified the shoes in which FOs were worn; extra-depth shoes with a wide toe-box [31, 35, 39] and forefoot-rockered extra-depth shoes with a wide toe-box were used [33]. A detailed description of the included studies is presented in Table 2.

**Table 2:** Characteristics of included studies and overview of FOs characteristics

Author (year)	Study design	Participant description	Intervention	Time	Outcome
*Region of interest - forefoot*					
Chalmers et al. 2000 [31]	randomized controlled clinical trial with a repeated measures design	**number** - *n* = 28 **setting** - occupational therapy department of hospital **diagnoses** - definitive diagnosis of RA **age (years)** - women: 60 (10) - men: 63 (2) *mean (SD)* **clinical characteristics** - subluxed MTP joints - bilaterally MTP joint pain	**Custom-made, semi-rigid (total-contact) FOs** - based on casts taken in a non-weight bearing position - constructed of semi-rigid material - addition of forefoot cushioning, and forefoot and hindfoot nickleplast posts *target of treatment* - support, stabilisation or correction of foot structures - cushioning (forefoot)	12 weeks for each intervention, separated by 2 week washouts	**foot pain** - VAS pain *(primary outcome)* **physical functioning** - 50 ft walking time, s* - RB* - TADL **patient satisfaction** - VAS treatment effectiveness - Nomination of the FOs of preference
			**Custom-made, soft (impression) FOs** - based on an impression in preheated plastazote during weight-bearing - constructed of soft materials - addition of metatarsal lifts *target of treatment* - support, stabilisation or correction of foot structures - cushioning (full length)		
			**Control intervention** - shoe-only		
Chang et al. 2011 [35]	controlled clinical trial with a repeated measures design (single session)	**number** - *n* = 19 **setting** - podiatric outpatient clinic of a hospital **diagnoses** - definitive diagnosis of RA **age (years)** - 58.6 (10.1) *mean (range)* **clinical characteristics** - forefoot pain - toe-deformities and/or hallux valgus	**Custom-made, semi-rigid (total-contact) FOs** - based on a foot impression (made in a foot impression box) while holding the subtalar joint at a neutral position - constructed of semi-rigid materials (cork) - addition of metatarsal support (cork) and cushioning material (full-length) *target of treatment* - support, stabilisation or correction of foot structures - cushioning, full length - forefoot plantar pressure reduction	1 month	**foot function** - In-shoe plantar foot pressure (peak pressure, pressure-time integral, mean force contact area) *(primary outcome)* **foot pain** - VAS pain **patient satisfaction** - Nomination of the FOs of preference
			**Custom-made, soft (impression) FOs** - based on impression in plastazote during weight-bearing (ADL 2–3 weeks) - constructed of soft materials - addition of metatarsal pad and arch support of EVA *target of treatment* - support, stabilisation or correction of foot structures - cushioning, full length - forefoot plantar pressure reduction		
			**Control intervention** - 7-mm flat EVA (40 Shore A hardness) FOs		
Gibson et al. 2014 [11]	controlled clinical trial with a repeated measures design (single session)	**number** - *n* = 16 **setting** - early arthritis clinic of a hospital **diagnoses** - definitive diagnosis of RA, > 2 years previously **age (years)** - 50.7 (8.4) * mean (range)* **clinical characteristics** - acquired and passively correctable pes plano valgus - with or without forefoot pain at MTP joints - orthotic naive	**Custom-made, semi-rigid (total-contact) FOs** - based on a plaster cast model of the foot using the subtalar joint neutral technique. - constructed of semi-rigid material (polypropylene) - optional adaptations (external rearfoot wedge control, arch height, forefoot cushioning) based on an algorithm of design rules. *target of treatment* - support, stabilisation or correction of foot structures - cushioning (forefoot) - forefoot plantar pressure reduction	7 days per intervention (without washout- periods)	**foot function** - Gait characteristics (rearfoot eversion, ankle internal moment, forefoot dorsiflexion, navicular height) *(primary outcome)* - In-shoe plantar foot pressure (forefoot peak pressure, midfoot contact area pressure-time integral, mean force contact area) *(primary outcome)* **patient satisfaction** - Likert scale (orthotic device comfort, orthotic device fit, self-reported efficacy, symptoms, activity levels) adverse events - minor and major
			**Custom-made, rigid (total-contact) FOs; CAD design using selective laser sintering** - the CAD design is based on a digitized plaster cast model of the foot using the subtalar joint neutral technique and an algorithm of design rules - manufactured using selective laser sintering using nylon-12 powder *target of treatment* - support, stabilisation or correction of foot structures - forefoot plantar pressure reduction		
			**Custom-made, semi-rigid (total-contact) FOs; CAD design using fused-deposition method** - the CAD design is based on a digitized plaster cast model of the foot using the subtalar joint neutral technique and an algorithm of design rules - manufactured using fused-deposition method using polylactide *target of treatment* - support, stabilisation or correction of foot structures - forefoot plantar pressure reduction		
			**Control intervention** - shoe-only		
Hodge et al. 1999 [36]	controlled clinical trial with a repeated measures design (single session)	**number** - *n* = 11 **setting** - University faculty of Health Science **diagnoses** - history of RA **age (years)** - 65 (49–82) *mean (range)* **clinical characteristics** - forefoot pain on shod weightbearing	**Custom-made, semi-rigid (total-contact) FOs** - based on the semi-weight bearing technique described by McPoil et al. (1989) using a latex rubber foot moulding board during moulding the EVA-material directly to the foot. - constructed of soft density, semi-rigid EVA - half-length FOs *target of treatment* - support, stabilisation or correction of foot structures - forefoot plantar pressure reduction	–	**foot function** - In-shoe plantar foot pressure (peak pressure, pressure-time integral, average pressure, time in mask) *(primary outcome)* - Gait characteristics (cadence) **foot pain** - VAS pain during standing - VAS pain during walking **patient satisfaction** - Nomination of the FOs of preference
			**Custom-made, semi-rigid (total-contact) FOs with additional metatarsal bars** - based on the semi-weight bearing technique described by McPoil et al. (1989) using a latex rubber foot moulding board during moulding the EVA-material directly to the foot. - constructed of soft density, semi-rigid EVA - addition of metatarsal bar (latex rubber, boomerang shape) - half-length FOs *target of treatment* - support, stabilisation or correction of foot structures - forefoot plantar pressure reduction		
			**Custom-made, semi-rigid (total-contact) FOs with additional metatarsal domes** - based on the semi-weight bearing technique described by McPoil et al. (1989) using a latex rubber foot moulding board during moulding the EVA-material directly to the foot. - constructed of soft density, semi-rigid EVA - addition of metatarsal dome (latex rubber, teardrop shape) - half-length FOs *target of treatment* - support, stabilisation or correction of foot structures - forefoot plantar pressure reduction		
			**Ready-made, soft FOs** - contoured soft density EVA FOs - half-length FOs *target of treatment* - support, stabilisation or correction of foot structures - forefoot plantar pressure reduction		
			**Control intervention** - shoe-only		
Jackson et al. 2004 [32]	randomized controlled trial with a repeated measures design (single session)	**number** - *n* = 10 **setting** - podiatry centre **diagnoses** - definitive diagnosis of RA **age (years)** *61 (32–79)* *mean (range)* **clinical characteristics** - forefoot pain on shod weightbearing	**Ready-made, soft FOs with additional metatarsal bars** - manufactured of expanded urethane foam with a hardness of 25 Shore A - addition of metatarsal square bar (latex foam, 29 Shore A) - full-length, contoured FOs *target of treatment* - support, stabilisation or correction of foot structures - forefoot plantar pressure reduction	–	**foot function** - In-shoe plantar forefoot pressure (peak pressure, pressure-time integral, stance time, contact area) *(primary outcome)* - Gait characteristics (cadence) **patient satisfaction** - Nomination of the FOs of preference
			**Ready-made, soft FOs with additional metatarsal domes** - manufactured of expanded urethane foam with a hardness of 25 Shore A - addition of metatarsal dome (latex foam, 29 Shore A) - full-length, contoured FOs *target of treatment* - support, stabilisation or correction of foot structures - forefoot plantar pressure reduction		
			**Control intervention** - shoe-only		
Tenten-Diepenmaat et al. 2016 [39]	quasi-experimental clinical trial with a repeated measures design (single session)	**number** - *n* = 45 **setting** - outpatient centre for rehabilitation and rheumatology **Diagnoses** - definitive diagnosis of RA **age (years)** *53 (13.5)* *mean (range)* **clinical characteristics** - RA related foot - indication for treatment with FOs	**Custom-made, semi-rigid (total-contact) FOs** - constructed of prefabricated orthotic devices, custom-moulded to the patient’s foot while using the functional suspension subtalar joint neutral position technique. - optional addition of varus-, valgus corrections, metatarsal bars, metatarsal domes, and/or cushioning material *target of treatment* - support, stabilisation or correction of foot structures - forefoot plantar pressure reduction	–	**foot function** - In-shoe plantar forefoot pressure (peak pressure, pressure-time integral) *(primary outcome)*
			**Custom-made, semi-rigid (total-contact) FOs, with adaptations using the feedback of in-shoe plantar pressure measurements** - custom-made, semi-rigid, total-contact FOs were adapted based on the feedback of in-shoe plantar pressure measurements - optional change or addition of varus-, valgus corrections, metatarsal bars, metatarsal domes, and/or cushioning material *target of treatment* - support, stabilisation or correction of foot structures - forefoot plantar pressure reduction		
*Region of interest - hindfoot*					
Gatt et al. 2016 [37]	controlled cross-over trial	**number** - n = 10 **setting** - rheumatology outpatient clinic at a general hospital **diagnoses** - definitive diagnosis of RA **age (years)** - 52.2 (9.1) *mean (SD)* **clinical characteristics** - subtalar and/or ankle joint pain ≥6 months - need of orthoses for biomechanical mal-alignment of the feet as per clinical practice	**Custom-made, semi-rigid (total-contact) FOs** - based on a cast (the positive casts were modified as outlined by Philips et al.) - constructed of semi-rigid material (subortolene) *target of treatment* - support, stabilisation or correction of foot structures	3 months per intervention, with a 2 week washout period in between	**foot pain** - FFI pain - RAI **physical functioning** - FFI disability - FFI limitation
			**Custom-made, soft (total-contact) FOs** - based on a cast (the positive casts were modified as outlined by Philips et al.) - constructed of low density EVA *target of treatment* - support, stabilisation or correction of foot structures		
*Region of interest - non-specified*					
Cho et al. 2008 [33]	randomized controlled trial	**number** - *n* = 42 (22 intervention-group, 20 control-group) **Setting** - university hospital **Diagnoses** - definitive diagnosis of RA **age (years)** - 48.7 (11.7) *mean (SD)* **clinical characteristics** - stable disease activity - foot pathology (forefoot or hindfoot)	**Custom-made, semi-rigid FOs** - consisting of a medial longitudinal arch support, medial heel post and metatarsal pad. *target of treatment* - support, stabilisation or correction of foot structures	6 months	**foot pain** - VAS pain *(primary outcome)*
			**Ready-made, soft FOs** - simple FOs - 6 mm plastazote *target of treatment* - cushioning, full length		
Pallari et al. 2010 [38]	quasi-experimental clinical trial with a repeated measures design (single session)	**number** - *n* = 7 **setting** - rheumatology outpatient clinic of a hospital **Diagnoses** - definitive diagnosis of RA **age (years)** *53.4 (29–68)* *mean (range)* **clinical characteristics** - current history of foot impairments	**Custom-made, semi-rigid (total-contact) FOs** - based on casts - mainly constructed of semi-rigid material - optional addition of cushioning material *target of treatment* - support, stabilisation or correction of foot structures	–	**foot function** - Gait characteristics (velocity, cadence, cycle-time, stride length) *(primary outcome)* **patient satisfaction** - VAS orthotic comfort - VAS orthotic fit
			**Custom-made, semi-rigid (total-contact) FOs; CAD design using selective laser sintering** - the CAD design is based on a weight or nonweight-bearing scan of the foot (in a subtalar joint neutral alignment) and on design rules - manufactured using selective laser sintering using nylon-12 powder *target of treatment* - support, stabilisation or correction of foot structures		
Rome et al. 2017 [34]	randomized controlled trial	**number** - *n* = 47 **setting** - rheumatology outpatient department **Diagnoses** - definitive diagnosis of RA **age (years)** *65 (49–82)* *mean (range)* **clinical characteristics** - history of foot pain	**Custom-made, semi-rigid (total-contact) FOs** - based on a cast taken of a neutral suspension plaster - constructed of semi-rigid material (50 Shore A) - optional addition of external medial posting correction - addition of cushioning material (full-length) *target of treatment* - support, stabilisation or correction of foot structures - cushioning	16 weeks	**foot pain** - FFI foot pain **physical functioning** - FFI disability - FFI functional limitation **Costs of FOs** - EQ 5D utility index (QUALYs) - mean cost of resource use
			**Custom-made, soft FOs** - constructed of a 6-mm breathable foam on a rubber-silicone-ethylene compound. - full-length FOs *target of treatment* - support, stabilisation or correction of foot structures - cushioning		

*VAS* visual analogue scale, *RB* Robinson Bashall Functional Assessment, *TADL* Toronto Activities of Daily Living Measure, *FFI* foot function index, *NRS* numeric rating scale, *EVA* Etylene Vinyl Acetate, *RAI* Ritchie Articular Index, *QUALYs* quality-adjusted life years, * performance based

### Methodological quality of included individual studies

Initial overall agreement on methodological quality scores was 96%. No consultation of the third reviewer was necessary to resolve disagreement. Methodological quality of included individual studies is presented in Table 3. Three studies were considered to be of high [31, 32, 34], six of fair [11, 33, 35, 36, 38, 39] and one of low quality [37].

**Table 3:** Methodological quality of included studies using the PEDro checklist

Reference	External validity (0–1)	Internal validity (0–10)										Total score	Quality
	1	2	3	4	5	6	7	8	9	10	11		
Chalmers et al. 2000 [31]	1	1	0	n/a	0	0	1	0	1	1	1	5/9 (56%)	High
Chang et al. 2011 [35]	0	0	0	n/a	0	0	0	1	1	1	1	4/9 (44%)	Fair
Cho et al. 2009 [33]	1	1	0	1	0	0	0	0	1	1	1	5/10 (50%)	Fair
Gatt et al. 2016 [37]	1	0	0	n/a	0	0	0	1	1	0	1	3/9 (33%)	Low
Gibson et al. 2014 [11]	1	0	0	n/a	0	0	0	1	1	1	1	4/9 (44%)	Fair
Hodge et al. 1999 [36]	0	0	0	n/a	0	0	0	1	1	1	1	4/9 (44%)	Fair
Jackson et al. 2004 [32]	1	1	0	n/a	0	0	0	1	1	1	1	5/9 (56%)	High
Pallari et al. 2010 [38]	1	0	0	n/a	0	0	0	1	1	1	1	4/9 (44%)	Fair
Rome et al. 2017 [34]	1	1	1	0	1	0	0	0	1	1	1	6/10 (60%)	High
Tenten-Diepenmaat et al. 2016 [39]	1	0	0	n/a	0	0	0	1	1	1	1	4/9 (44%)	Fair

High quality = study quality percentage ≥ 55–100%. Fair quality = study quality percentage ≥ 35- < 55%. Low quality = study quality percentage < 35%. n/a = not applicable

### Comparisons in treatment with FOs

**Fig. 2:**
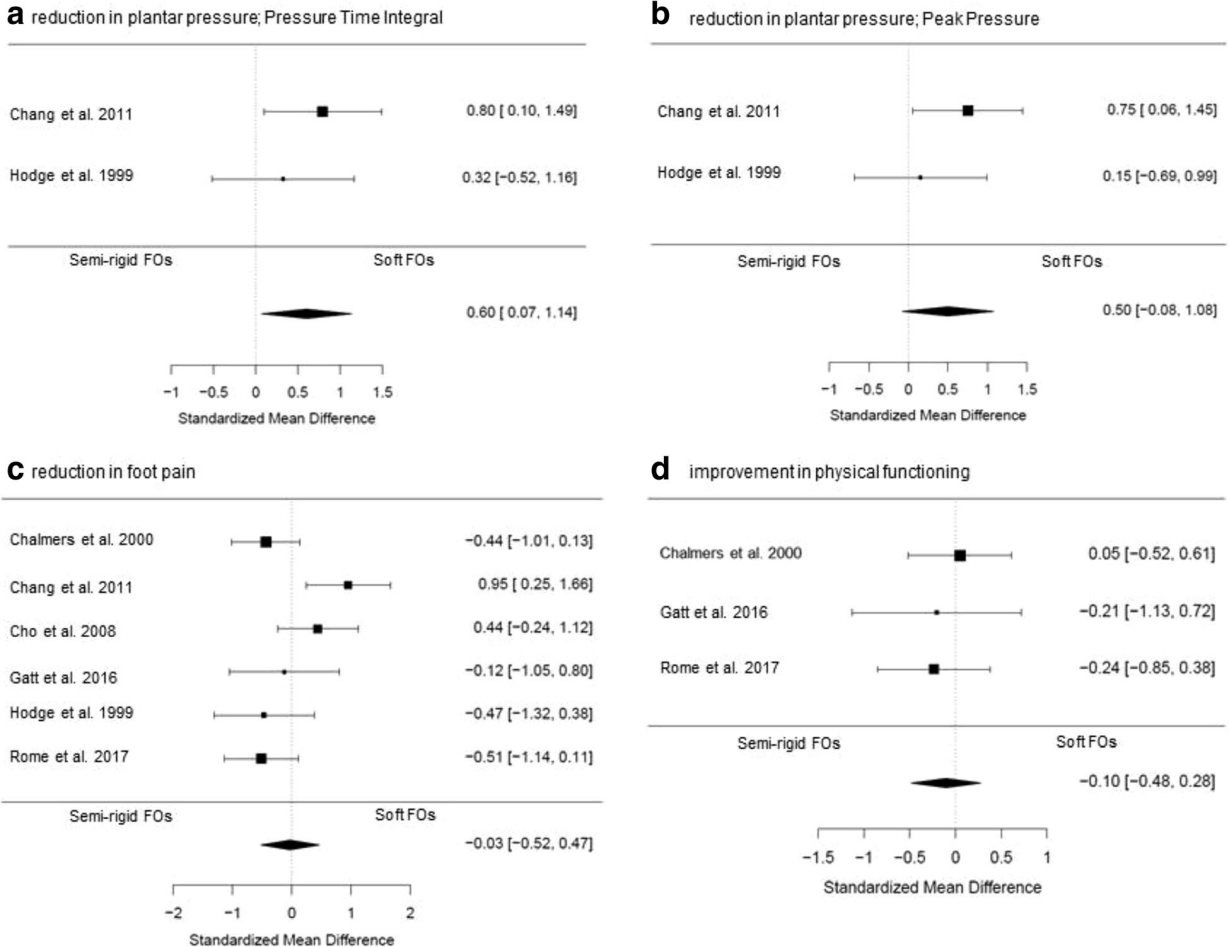
Forest plots of data pooling for the effect of semi-rigid FOs versus soft FOs on (**a**) foot function expressed as Pressure Time Integral, (**b**) foot function expressed as Peak Pressure, (**c**) foot pain, and (**d**) physical functioning

**Fig. 3:**
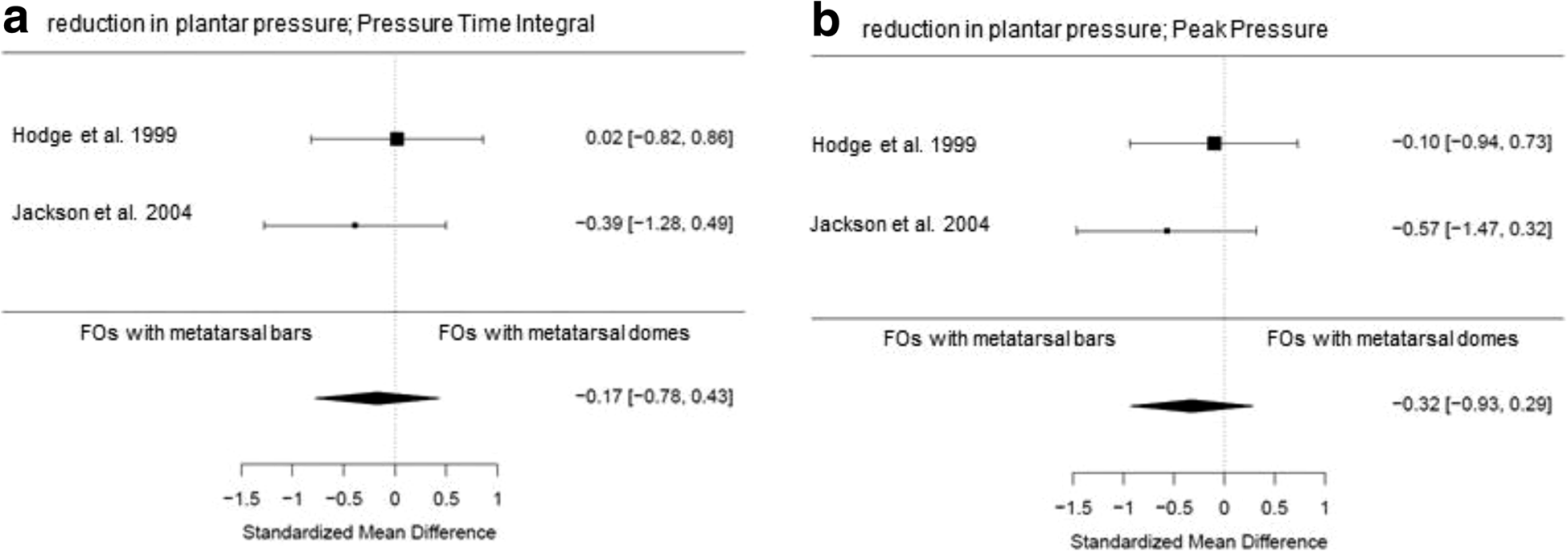
Forest plots of data pooling for the effect of FOs with metatarsal bars versus FOs with metatarsal domes on (**a**) foot function expressed as Pressure Time Interval, (**b**) foot function expressed as Peak Pressure

Different FOs characteristics and different construction methods for manufacturing FOs were identified in the included studies, allowing comparisons of effectiveness. Meta-analyses are presented in Figs. 2 and 3. Subgroup meta-analyses are shown in Additional file 2. When meta-analysis was not possible, qualitative data-analysis was performed as shown in Additional file 3. Although subgroup meta-analyses on study quality and shoe-condition were planned a priori, these analyses were not possible due to an insufficient number of studies.

### Characteristics of FOs

Different FOs characteristics were identified concerning (i) materials used for manufacturing the shell (base-frame) of FOs, (ii) type of FOs, and (iii) modifications applied to the FO-shell. Concerning materials used for manufacturing the shell of FOs a distinction could be made between soft (cushioning effect) [31–35, 37] and semi-rigid [11, 31, 33–39] materials. Semi-rigid FOs are manufactured of materials with a stiffness aimed to provide control of the position of the feet during weight-bearing. A comparison was made for the effect of ‘semi-rigid FOs *versus* soft FOs’ [31, 33–37]. Within this comparison four subgroups were identified. Two subgroups concerned FO-type, in which the comparisons ‘custom-made (semi-rigid) FOs *versus* ready-made (soft) FOs’ [33, 36] and ‘total-contact (semi-rigid) FOs *versus* non-total contact (soft) FOs’ [31, 34–36] were investigated. Furthermore, one subgroup was identified with the forefoot as region of interest for treatment [31, 35, 36], and in one subgroup the effect of treatment was measured after > 1 month of wearing FOs (in contrast to immediate effect) [31, 33–35, 37]. For type of FOs a distinction could be made between custom-made FOs [31, 33–40] and ready-made (i.e. off-the-shelf or over-the counter) FOs [32, 33, 36], and between total-contact [11, 31, 34–39] and non-total-contact FOs [31–36]. For modifications applied to the FO-shell a distinction could be made between metatarsal bars [32, 36] and metatarsal domes [32, 36]. A comparison was made for the effect of ‘FOs with metatarsal bars *versus* FOs with metatarsal domes’ [32, 36].

#### Comparative effectiveness of semi-rigid FOs versus soft FOs

Six included studies (two of high (75 participants) [31, 34], three of fair (72 participants) [33, 35, 36] and one of low quality (10 participants) [37]) investigated the effect of treatment with FOs constructed of a semi-rigid shell versus soft FOs constructed of a soft shell. Pooled scores showed a medium, statistically significant, immediate effect for reduction of forefoot plantar pressure-time integral (PTI) in favor of treatment with soft FOs (SMD 0.60, 95% CI 0.07–1.14; *P* = 0.03; 28 participants; Fig. 2a). A similar effect was found for forefoot plantar peak pressure (PP), although not statistically significant (SMD 0.50, 95% CI -0.08 – 1.08; *P* = 0.09; 28 participants; Fig. 2b). For foot pain, pooled scores (SMD 0.03, 95% CI -0.47 – 0.52; *P* = 0.91; 157 participants; Fig. 2c) and subgroup meta-analyses (Additional file 2) showed no effect in favor of treatment with one type of FOs over the other, as well as for pooled scores for physical functioning (SMD -0.10, 95% CI -0.48 – 0.28; *P* = 0.59; 54 participants; Fig. 2d). Funnel plots were constructed for the analyses on foot pain and physical functioning (Additional file 4). Limited evidence for publication bias was found, since for the smaller studies treatment effects are spread evenly on both sides of the average (as shown in Additional file 4). Qualitative data-syntheses resulted in inconclusive evidence for one type of FOs over the other on the secondary outcomes compliance, costs of FOs and patient satisfaction.

#### Comparative effectiveness of FOs with metatarsal bars versus FOs with metatarsal domes

Two included studies (one of high quality (10 participants) [32] and one of fair quality (11 participants) [36]) investigated the effect of different types of metatarsal support (FOs with metatarsal bar versus FOs with metatarsal dome) in the treatment of forefoot problems. Pooled scores showed a small, immediate, not statistically significant, effect in favor of FOs with metatarsal bars for reduction of forefoot plantar pressure (PTI (SMD -0.17, 95% CI -0.78 – 0.43; *P* = 0.58; 22 participants; Fig. 3a) and PP (SMD -0.32, 95% CI -0.93 – 0.29; *P* = 0.30; 22 participants; Fig. 3b)). Qualitative data-syntheses resulted in inconclusive evidence for one type of FOs over the other on the primary outcome foot pain and the secondary outcome patient satisfaction.

### Construction method for FOs

Within the included studies various methods were used for manufacturing custom-made FOs; selective laser sintering [11, 38] and standard methods for custom-molding of material, i.e. directly to the foot [36, 39], or by using an impression- or plaster cast model [11, 31, 34, 35, 37, 38]. A comparison could be made for the effect of ‘selective laser sintered FOs *versus* standard custom-made FOs’ [11, 38].

#### Comparative effectiveness of selective laser sintered FOs versus standard custom-made FOs

In two studies (of fair quality (23 participants) [11, 38]) the feasibility and outcomes on foot function of custom-made FOs manufactured by using sophisticated construction methods were compared to standard methods. Gibson et al. [11] reported more immediate forefoot plantar pressure reduction in favor of treatment with selective laser sintered FOs, although not statistically significant. Pallari et al. [38] reported a slightly (non-tested) faster cadence in favor of treatment with standard custom-made FOs. Qualitative data-syntheses resulted in inconclusive evidence for foot function measured with either plantar pressure or gait parameters between the different construction methods. Furthermore, inconclusive evidence was found for one type of FOs over the other on the secondary outcome patient satisfaction.

## Discussion

To our knowledge, this is the first published systematic review investigating the comparative effectiveness of FOs in patients with RA. The included studies showed a distinction in FOs characteristics (concerning materials, type and modifications) and construction methods for custom-made FOs (sophisticated versus standard techniques). The target of treatment with FOs was mostly reduction of forefoot plantar pressure or forefoot pain. A medium effect for the (immediate) reduction of forefoot plantar pressure was found in favor of treatment with soft FOs compared to semi-rigid FOs. Other comparisons concerning characteristics of FOs or construction methods resulted in non-significant effects or inconclusive evidence for one type of FOs over the other for both primary and secondary outcomes.

It is known that custom-made FOs are more effective in reducing forefoot plantar pressure and pain than placebo FOs [9, 14]. However, the comparative effectiveness has not yet been summarized. The findings of the present study show that soft FOs may lead to more (immediate) forefoot plantar pressure reduction than semi-rigid FOs (based on a sample size of 28 participants). Pooled scores on foot pain showed no beneficial effect of treatment with soft FOs over semi-rigid FOs. This could possibly be explained by the already small effects on foot pain of treatment with custom-made FOs in general [9, 14], making the potential for demonstrating a beneficial effect between different types of custom-made FOs difficult, especially in case of small sample sizes. Cultural differences may also have contributed to this result. The forest plot of the pooled pain scores (Fig. 2b) shows inconsistent findings across the included studies, for one type of FOs over the other. Four (out of six) studies were performed in the Western parts of the world and showed all a beneficial effect of semi-rigid FOs over soft FOs [31, 34, 36, 37]. The other two studies were performed in Asian countries and showed contrary findings [33, 35]. It is not known whether differences in body structure or shoe wearing habits could explain this difference. Finally, an explanation could be that reduction of plantar pressure may not be the primary mediator between FOs treatment and foot pain. For example, the study of Hodge et al. (fair quality, 11 participants) showed more forefoot plantar pressure reduction after using soft FOs, but more pain reduction was reached by using semi-rigid FOs [36]. To further clarify the mechanism behind the effect of FO’s, future research should assess the kinematic and kinetic response to treatment with FOs [41]. In RA patients with early and painful deformity of the rearfoot, correction of deformity and optimization of function of the ankle joint complex were detected by measuring three-dimensional kinematics by using an electromagnetic tracking system after the long-term use of custom-made FOs [42]. Further insight in the kinematic and kinetic response to the use of FOs, as well as the association with clinical outcomes in patients with RA and (fore-) foot problems is required. A clinical trial on this topic is planned by researchers in Denmark (ClinicalTrials.gov (accessed October 22th 2018); Trial Identifier NCT03561688).

More research on the comparative effectiveness of FOs is necessary before firm conclusions can be drawn. Overall, few high-quality studies with small sample sizes were included in the present review. Due to a limited number of studies investigating the outcomes of interest most of the performed qualitative data-analyses resulted in inconclusive evidence. For example, cost effectiveness between different types of FOs was investigated in only one included study (of high quality, 47 participants) [34]. Rome et al. found that custom-made, semi-rigid (total-contact) FOs were far more expensive to manufacture with no significant cost per Quality Adjusted Life Years (QALY) gain, in comparison to custom-made, soft FOs [34]. Due to the included study designs, between-group results of different types of FOs were presented in only a part of the included studies. Some studies reported results of different kinds of FOs (with a presumed therapeutic effect) but were not designed to compare the effect of those [31–33, 35–37]. These studies met our inclusion criteria, but provided limited information with regard to the comparative effectiveness of the different FOs. Furthermore, most of the included studies investigated the immediate effect on foot pain or the immediate mode of action on plantar pressure/gait alteration (in a laboratory setting instead of real-life). Future research with a follow-up of > 6 months [10] is necessary. An acclimation period of wearing FOs, especially semi-rigid FOs, is needed before the final result on pain will be reached [10, 31]. Long-term follow-up is also needed to identify the potential role of treatment with semi-rigid FOs (aimed at controlling the position of the feet during weight-bearing) in delaying progression of foot symptoms in patients with early RA [43–45].

The present study provides a first step in gaining insight in the effectiveness of different FOs characteristics. Future research could focus on the development of practice recommendations for prescribing/designing FOs with optimal characteristics for (delaying progression of) specific RA-related foot problems. Therefore, definitive high quality RCTs, with adequate sample sizes and long-term follow-up, are needed to investigate the comparative (and cost-) effectiveness of different types of FOs for the treatment of RA-related foot problems. In anticipation of more up-to-date insights, prescribing custom-made (total-contact) FOs constructed of a semi-rigid shell with soft/cushioning material underneath the forefoot might be the most optimal approach in the treatment of RA-related foot problems, as suggested by recently published expert-based recommendations by our group [45]. The use of soft material underneath the forefoot is supported by the results of the present review. Furthermore, a stepped-care approach was suggested [45]. Based on specific diagnostical outcomes (conservative) stepped care for RA-related foot problems can consist of; i) advice on-over-the-counter shoes, ii) ready-made FOs, iii) custom-made FOs, and iv) therapeutic shoes. Further research on this stepped-care approach is necessary. Gallaher et al. announced upcoming trial-evidence on custom-made FOs versus ready-made FOs in patients with RA, by publishing their study-protocol [46]. Moreover, further development of sophisticated construction methods may be important for uniformity and (cost-) efficiency in designing custom-made FOs. Gibson et al. [11] and Pallari et al. [38] showed that selective laser sintering is a feasible method for manufacturing FOs with a significant clinical potential.

This study has some limitations. First, publication bias cannot be ruled out. The majority of the included studies were small-sample studies. Inspection of funnel plots, however, showed limited evidence of publication bias. Further, the search strategy did not include unpublished literature, such as theses and conference proceedings. It may be that not all studies carried out have actually been published*.* Second, there is large heterogeneity in study designs and outcome measures of the included studies. Furthermore, variation may exist between FOs within the different categories (concerning FOs characteristics and construction methods). Third, due to the small evidence base we chose to aggregate the outcome on foot pain of studies with no or differing follow-up time within meta-analyses. In a subgroup analysis, we studied the impact of ≥1 month follow-up, showing no effect (SMD 0.05, *p* > 0.05) on foot pain (Additional file 2). Fourth, studies using placebo FOs were excluded in the present review. However, the characteristics of placebo FOs varied across these studies [47–50] indicating that the definition of placebo FOs is not yet established.

## Conclusions

Foot orthoses made of soft materials may lead to more (immediate) forefoot plantar pressure reduction compared to foot orthoses constructed of semi-rigid materials. Definitive high quality RCTs, with adequate sample sizes and long-term follow-up, are needed to investigate the comparative (cost-) effectiveness of different kinds of foot orthoses for the treatment of foot problems related to rheumatoid arthritis.

## Additional files

Additional file 1: Search strategy. (PDF 84 kb)

Additional file 2: Forest plots of data pooling for the effect of semi-rigid FOs versus soft FOs on pain in the subgroups: (a) custom-made FOs versus ready-made FOs, (b) total-contact FOs versus non-total contact FOs, (c) forefoot region of interest, and (d) treatment effect after ≥ 1 month. (PDF 141 kb)

Additional file 3: Qualitative data-syntheses. (PDF 209 kb)

Additional file 4: Funnel plots for the comparison ‘semi-rigid FOs versus soft FOs’ on the outcomes (a) foot pain and (b) physical functioning. (PDF 116 kb)

## Abbreviations

95% CI: 95% Confidence Interval
CAD: Computer-Aided Design
CAM: Computer-Aided Manufacturing
EVA: Etylene Vinyl Acetate
FFI: Foot Function Index
FHSQ: Foot Health Status Questionnaire
FOs: Foot Orthoses
HRQoL: Health Related Quality of Life
PEDRO scale: Physiotherapy Evidence Database scale
PP: Peak Pressure
PRISMA: Preferred Reporting Items for Systematic Reviews and Meta-Analyses
PTI: Pressure Time Integral
QALY: Quality Adjusted Life Years
RA: Rheumatoid Arthritis
RAI: Ritchie Articular Index
RB: Robinson Bashall Functional Assessment
RCT: Randomized Controlled Trial
SMD: Standardized Mean Difference
TADL: Toronto Activities of Daily Living Measure
VAS: Visual Analogue Scale
