# Supplementary material for: Systematic review on the comparative effectiveness of foot orthoses in patients with rheumatoid arthritis
Source: J Foot Ankle Res. 2019 Jun 13;12:32. doi: 10.1186/s13047-019-0338-x (PMC6567436; doi:10.1186/s13047-019-0338-x)
Supplement: Supplementary file 1 — Search strategy. (PDF 84 kb) [file 13047_2019_338_MOESM1_ESM.pdf]

## Additional file 1. Search strategy

A two-way search strategy was employed using “rheumatoid arthritis”, “foot orthoses” with “foot” and related synonyms of these terms.

The following database search strategy for PubMed was used:

("Arthritis, Rheumatoid"[Mesh] **OR** rheumatoid arthritis [tiab]) **AND** ("foot orthoses"[Mesh] **OR** "orthotic devices"[Mesh] **OR** "inlays"[Mesh] **OR** orthos*[tiab] **OR** orthotic*[tiab] **OR** inlay*[tiab] **OR** insert*[tiab] **OR** insole*[tiab]) **OR** foot arch support [tiab]) **AND** ("foot"[Mesh] **OR** "Foot Bones"[Mesh] **OR** "Ankle"[Mesh] **OR** foot[tiab] **OR** feet[tiab] **OR** ankle[tiab] **OR** rearfoot[tiab] **OR** hindfoot[tiab] **OR** midfoot[tiab] **OR** "Forefoot, Human"[Mesh] **OR** forefoot[tiab] **OR** tarsal[tiab] **OR** "Talus"[Mesh] **OR** talus[tiab] **OR** "Calcaneus"[Mesh] **OR** calcan*[tiab] **OR** subtalar[tiab] **OR** sinus tars*[tiab] **OR** talonavicular*[tiab] **OR** "Metatarsus"[Mesh] **OR** metatarsal*[tiab] **OR** metatarsophalang*[tiab] **OR** "Heel"[Mesh] **OR** heel[tiab] **OR** "Fibula"[Mesh] **OR** fibula[tiab] **OR** "Tibia"[Mesh] **OR** tibia[tiab] **OR** "Toes"[Mesh] **OR** toe*[tiab] **OR** phalang*[tiab] **OR** "Hallux"[Mesh] **OR** hallux[tiab])
