# Supplementary material for: Systematic review on the comparative effectiveness of foot orthoses in patients with rheumatoid arthritis
Source: J Foot Ankle Res. 2019 Jun 13;12:32. doi: 10.1186/s13047-019-0338-x (PMC6567436; doi:10.1186/s13047-019-0338-x)

Additional file 2. Forest plots of data pooling for the effect of semi-rigid FOs *versus* soft FOs on pain in the subgroups: (a) custom-made FOs versus ready-made FOs, (b) total-contact FOs versus non-total contact FOs, (c) forefoot region of interest, and (d) treatment effect after ≥1 month.

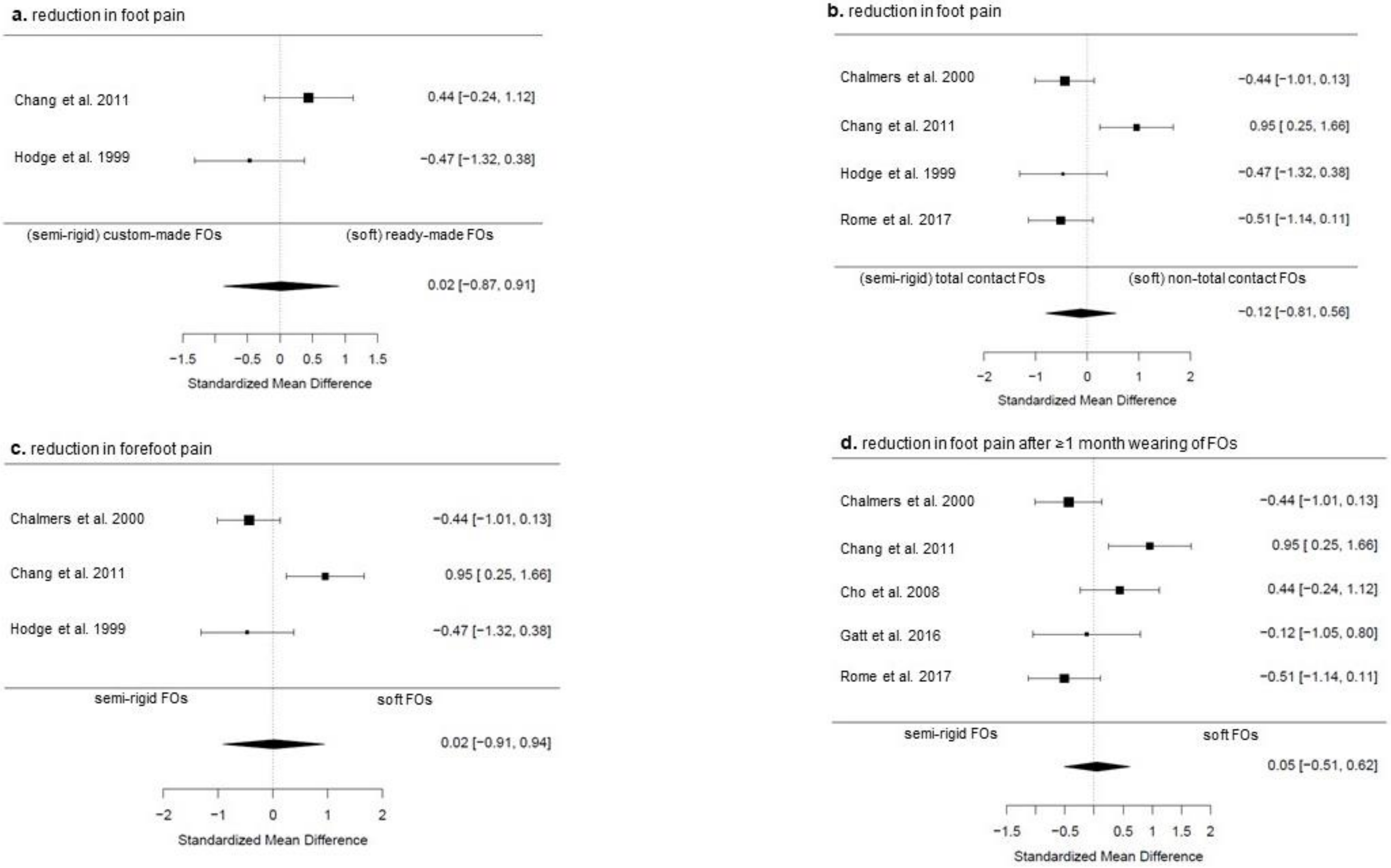

Supplement: Supplementary file 2 — Forest plots of data pooling for the effect of semi-rigid FOs versus soft FOs on pain in the subgroups: (a) custom-made FOs versus ready-made FOs, (b) total-contact FOs versus non-total contact FOs, (c) forefoot region of interest, and (d) treatment effect after ≥ 1 month. (PDF 141 kb) [file 13047_2019_338_MOESM2_ESM.pdf]
