# Supplementary material for: Systematic review on the comparative effectiveness of foot orthoses in patients with rheumatoid arthritis
Source: J Foot Ankle Res. 2019 Jun 13;12:32. doi: 10.1186/s13047-019-0338-x (PMC6567436; doi:10.1186/s13047-019-0338-x)
Supplement: Supplementary file 3 — Qualitative data-syntheses. (PDF 209 kb) [file 13047_2019_338_MOESM3_ESM.pdf]

Additional file 3. Qualitative data-syntheses

Outcome	Level of evidence	Results	References
semi-rigid FOs (a) versus soft FOs (b)			
compliance	inconclusive	*0.26 hour more wearing time a day in favor of treatment with intervention a; P = n/a*	Chalmers et al. 2000 (31)
costs of FOs	inconclusive	*-0.03 (95% CI -0.08 – 0.03) QALYs in favor of treatment with intervention a; P = 0.46*	Rome et al. 2016 (34)
patient satisfaction	inconclusive	*a and b were both nominated as preferred FOs by 11 (out of 24) participants; P = n/a*	Chalmers et al. 2000 (31)
		82% more participants nominated intervention b as preferred FOs; P = n/a	Chang et al. 2011 (35)
		20% more participants nominated intervention a as preferred FOs; P = n/a	Hodge et al. 1995 (36)
custom-made (semi-rigid) FOs (a) versus ready-made (soft) FOs (b)			
foot function, construct plantar pressure	inconclusive	*0.99 (16%) lower forefoot plantar pressure (PTI) in favor of treatment with intervention b; P = n/a*	Hodge et al. 1995 (36)
patient satisfaction	inconclusive	*20% more participants nominated intervention a as preferred FOs; P = n/a*	Hodge et al. 1995 (36)
total-contact (semi-rigid) FOs (a) versus non-total-contact (soft) FOs (b)			
costs of FOs	inconclusive	*-0.03 (95% CI -0.08 – 0.03) QALYs in favor of treatment with intervention a; P = 0.46*	Rome et al. 2016 (34)
patient satisfaction	inconclusive	*a and b were both nominated as preferred FOs by 11 (out of 24) participants; P = n/a*	Chalmers et al. 2000 (31)
		82% more participants nominated intervention b as preferred FOs; P = n/a	Chang et al. 2011 (35)
		20% more participants nominated intervention a as preferred FOs; P = n/a	Hodge et al. 1995 (36)

selective laser sintered FOs (a) versus standard custom-made FOs (b)			
foot function, plantar pressure	inconclusive	*9.3 (13.6) lower medial forefoot plantar pressure (PP) in favor of treatment with intervention a; P = 1.00*	Gibson et al. (11)
foot function, gait	inconclusive	*2 more steps per minute (cadence) in favor of treatment with intervention b; P = n/a*	Pallari et al. 2010 (38)
patient satisfaction	inconclusive	*2.4 higher VAS fit of FOs in favor of treatment with intervention b; P ≥0.05*	Pallari et al. 2010 (38)
FOs with metatarsal bars (a) versus FOs with metatarsal domes (b)			
foot pain	inconclusive	*7 mm on VAS-score less pain in favor of treatment with intervention b*	Hodge et al. 1995 (36)
patient satisfaction	inconclusive	*intervention a was nominated by 30% of participants and intervention b by 50% as preferred FOs*	Hodge et al. 1995 (36)
		intervention a was nominated by 30% of participants and intervention b by 70% as preferred FOs	Jackson et al. 2004 (32)

FOs = foot orthoses. VAS = visual analogue scale. QUALYs = quality-adjusted life years. PTI = pressure time integral. PP = peak pressure.
