# Supplementary material for: Systematic review on the comparative effectiveness of foot orthoses in patients with rheumatoid arthritis
Source: J Foot Ankle Res. 2019 Jun 13;12:32. doi: 10.1186/s13047-019-0338-x (PMC6567436; doi:10.1186/s13047-019-0338-x)

Additional file 4. Funnel plots for the comparison 'semi-rigid FOs *versus* soft FOs' on the outcomes (a) foot pain and (b) physical functioning

a. foot pain

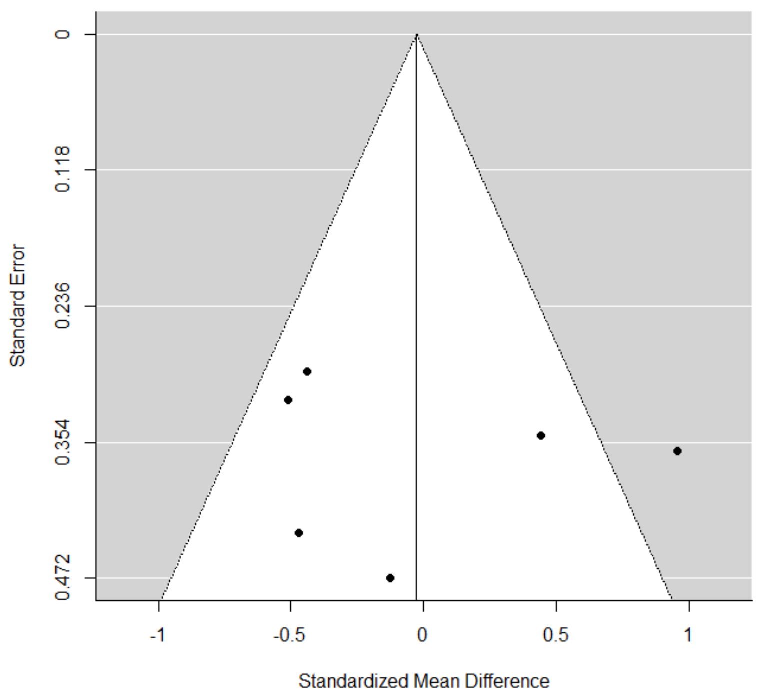

b. physical functioning

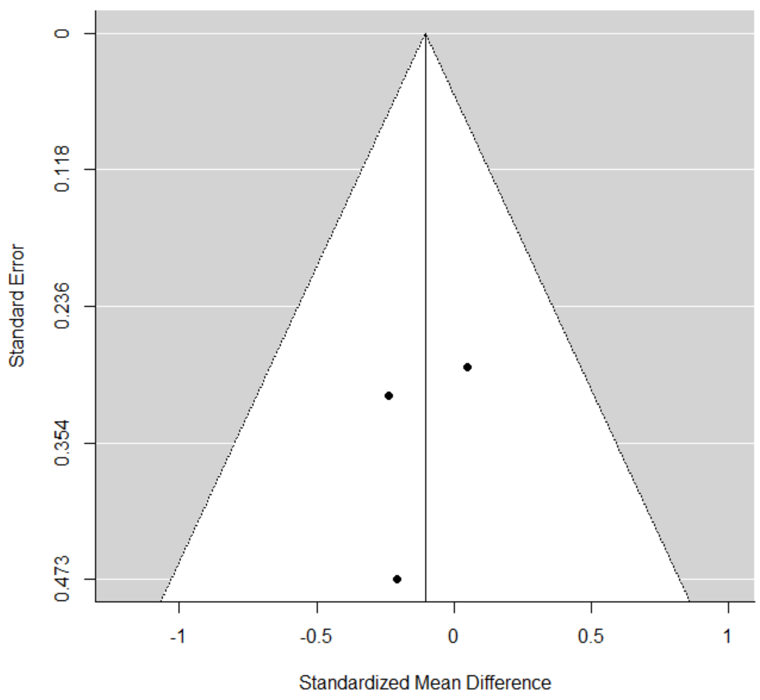

Supplement: Supplementary file 4 — Funnel plots for the comparison ‘semi-rigid FOs versus soft FOs’ on the outcomes (a) foot pain and (b) physical functioning. (PDF 116 kb) [file 13047_2019_338_MOESM4_ESM.pdf]
